# Nurses’ Experiences of Caring for Patients with Dementia in Supportive Treatment and Nursing Hospitals in Lithuania: A Qualitative Study

**DOI:** 10.3390/nursrep16040124

**Published:** 2026-04-08

**Authors:** Agnė Jakavonytė-Akstinienė, Karolina Adomavičiūtė

**Affiliations:** Department of Midwifery and Nursing, Institute of Health Sciences, Medical Faculty of Vilnius University, M. K. Čiurlionio St. 21, LT-03101 Vilnius, Lithuania

**Keywords:** nurses, experiences, dementia patients, patients, care, needs

## Abstract

**Background:** Dementia is one of the most common diseases of the elderly worldwide. Sharing experiences of caring for patients with dementia with other carers is essential to improve the quality of care, promote better outcomes, and learn from others. **Aim:** to explore nurses’ experiences of working with patients with dementia in Lithuanian supportive treatment and nursing hospitals. **Methods:** A qualitative descriptive design was employed in this study, with data collected through semi-structured interviews. Nurses with direct experience caring for patients with dementia in supportive treatment and nursing hospitals were recruited through purposive sampling. This sampling strategy was chosen to ensure that participants could provide rich, contextual, and experience-based insights into the phenomenon under investigation. Open-ended questions were divided into three themes: 1. Identifying nursing needs. 2. Care for people with dementia. 3. Patient behavior management and situation management. To ensure methodological rigor and transparency, the Consolidated Criteria for Reporting Qualitative Research (COREQ) were applied throughout the study’s planning, data collection, and analysis processes. **Results:** Nine nurses working in three different Lithuanian hospitals participated in the study. Theme 1: respondents reported that the needs of patients with dementia depend on their previous lifestyle and hobbies, as well as on essential physiological needs such as eating and drinking, bathing and personal hygiene, and the absence of pain. Theme 2: All participants emphasized that ensuring a safe environment is crucial for people with dementia. Theme 3: When faced with inappropriate patient behaviour, nurses attempt to calm the patient, speak gently, provide distraction, or, when necessary, temporarily separate the patient from others. Additional actions include administering medication and stabilizing the patient. Overall, these findings illustrate that dementia care requires continuous emotional presence, situational judgment, and adaptation to each patient’s individual needs. **Conclusions:** Patients with dementia require highly individualized care focused on nutrition, hygiene, pain control, and communication. Nurses’ daily activities centered on essential bodily care, medication management, and mobility support to maintain safety and prevent complications.

## 1. Introduction

One of the most common diseases in older people is dementia. According to the World Health Organisation (WHO), 55 million people worldwide have this syndrome. This number is increasing daily, with a projection of 78 million patients diagnosed with dementia by 2030 and as many as 139 million by 2050 [1]. The WHO considers dementia a global public health priority, aiming to improve the lives of people with dementia, their caregivers, and families by reducing the impact of dementia on them, as well as on communities and countries [2].

Studies examining hospital-based healthcare professionals consistently highlight gaps in both dementia-related knowledge and attitudes towards dementia care. Zhao et al. (2022) reported that healthcare professionals working in hospital settings demonstrated notable deficits in dementia-related knowledge and exhibited relatively low levels of positive attitudes towards dementia care [3]. A systematic review by Evripidou et al. (2019) found that nurses lacked knowledge, communication skills, management strategies, and confidence in providing care [4]. Additionally, Addis and Evans (2025) found that nurses working in acute care settings require further education and training to enhance their knowledge and skills in dementia care [5]. Strøm, Engedal, and Andreassen (2019) found that the overall mean knowledge score (19.5 out of 30) suggests that the participants have limited knowledge about dementia [6]. Besides, Dookhy and Daly (2021) found that the key challenges reported by nurses encompassed the acute hospital environment, a range of emotional difficulties, and a heightened need for advanced knowledge [7].

As dementia progresses and becomes more advanced, many more care needs become evident. Some of the most critical physiological needs are feeding, personal hygiene, and helping the person shower, bathe, shave, and brush their teeth, partially or fully. Regular defecation and urination are also important. Dressing is also an essential need for people with dementia, where the person can dress themself or needs help and guidance on what clothes to wear [8]. It also highlights the need for chronic pain relief, an essential factor for a better quality of life [9]. Although patients are usually confined to bed in the more severe stages of the disease, it is essential to ensure that they can move around as much as possible [8]. It is necessary to provide for emotional and physical needs.

Studies in Chile [10] and Poland [11] have shown that the most common needs of people with dementia are daily occupation and communication with others. One need for emotional well-being is security. People with dementia are also prone to wandering, to becoming active both day and night, and are thus prone to disappearance or injury. A study found that as many as 56% of nursing patients wandered and were at risk of leaving, underscoring the importance of continuous patient monitoring [12]. Spiritual support, which covers spiritual and religious needs, is necessary for people with dementia. When dealing with serious illness, grief, or pain, it can help patients regain hope and meaning in life [13]. The care of patients with dementia runs the risk of focusing only on the diagnosis. It should be stressed that each patient is an individual with their own history, hobbies, and personality traits. Time and good communication skills are needed to ensure that the care and needs of each individual are fully met [14].

Staying anger-free, ensuring good nutrition, pain management, providing a safe environment, and maintaining personal hygiene are the basic nursing needs when caring for patients with dementia. Anger, agitation, aggressiveness, insults, and other inappropriate reactions are perhaps the most common reactions nurses face in dementia wards. Nurses must know non-pharmacological and pharmacological ways to calm the patient [15]. Recommended actions that nurses can take to calm the patient include redirecting attention, playing video footage on TV, removing annoying objects, keeping the patient calm, and inviting another member of staff [16]. Another challenge is that nurses spend much time ensuring adequate nutrition, as dementia can cause problems with swallowing and memory. The patient needs continuous monitoring, and a special diet should be selected to prevent choking. Often, even after eating, the patient may say that he or she is hungry because he or she has forgotten that he or she has eaten and does not feel full [17]. Identifying pain is very important for patients with dementia, too. Verbal communication is unreliable for pain assessment, and in the case of cognitive impairment, the patient may not be able to identify the severity of the pain, so we have to rely on non-verbal cues, such as moaning, shouting, fidgeting, body language, and facial expressions. The dose of medication is also an essential factor, followed by monitoring for pain relief but not respiratory depression from overdose [18]. Medicines administered should also be safely placed out of patients’ reach [19]. Patients often lose coordination and fall due to a small space, lack of self-protection, inability to follow instructions, or agitation [20]. Depending on the stage of dementia, eventually, the patient stops taking care of their hygiene. Nursing care is needed to assist with bathing, dressing, washing, brushing teeth, nail hygiene, and hair care [21]. The most challenging activity is brushing teeth, as patients often become frustrated and aggressive when another person performs it due to oral mucosal sensitivity [8].

Nurses caring for patients with dementia face insufficient autonomy and ethical challenges. Gjellestad et al. (2022) found that nurses’ decision-making is reduced when patients resist care [22]. Their judgment is shaped by contextual factors and a strong commitment to avoid forced treatment. A persistent challenge is maintaining shared decision-making while balancing the risk of serious harm for home-dwelling people with dementia [22]. Besides, Pritchard and Vieira-Moreno (2025) stated that nursing people with dementia involves navigating ethical dilemmas, such as therapeutic lying, doll therapy, dementia pets, covert medication, and the use of monitoring technologies [23]. Each dilemma requires careful consideration and balancing against biomedical ethical principles [23]. Evidence shows that sharing negative and positive experiences enhances motivation to improve professional performance, collaboration, access to information, and nurse well-being [24].

In Lithuania, supportive treatment and nursing services include symptomatic treatment and nursing services for people of any age whose health condition meets the conditions set out in the Description, when there is a precise diagnosis of the disease and active treatment is not necessary, and are divided into general nursing, nursing for patients with dementia, and nursing for patients in a vegetative state. Nursing care for patients with dementia is a supportive treatment and nursing service provided to persons with dementia who have been diagnosed with general functional impairment due to somatic diseases and disorders, and who are entirely or almost entirely dependent on the help of others in their daily lives [25]. In Lithuania, supportive treatment and nursing hospitals are characterized by a distinct organizational structure, limited human resources, and varying levels of staff preparedness, yet no studies to date have examined how these conditions shape nurses’ experiences in providing daily dementia care. This knowledge gap limits a clear understanding of the practical and organizational factors that shape care quality in the Lithuanian context. Aim to explore nurses’ experiences of working with patients with dementia in Lithuanian supportive treatment and nursing hospitals.

## 2. Materials and Methods

### 2.1. Study Design and Setting

This study employed a qualitative descriptive design, well-suited to exploring healthcare professionals’ experiences in contexts with limited prior research. A qualitative descriptive approach enables researchers to remain close to participants’ accounts and to generate findings that reflect their perspectives without imposing a predefined theoretical or conceptual framework. Given that dementia care in Lithuanian supportive treatment and nursing hospitals is an understudied area, a qualitative descriptive design allowed themes to emerge directly from the data.

A qualitative study was conducted. The Consolidated Criteria for Reporting Qualitative Research (COREQ) were followed to ensure the study’s methodology was accurate and transparent.

The study was planned to interview nurses from different supportive treatment and nursing hospitals to make it more comprehensive. Between 28 February and 8 March 2023, with the permission of the three facilities, the wards’ senior nurses were contacted to help identify nurses working with dementia patients. Three nurses were from X Supportive Treatment and Nursing Hospital, three from Z Supportive Treatment and Nursing Hospital, and three from facility Y.

### 2.2. Study Participants

A purposeful sampling approach was used to recruit nurses with direct experience in caring for patients with dementia in supportive treatment and nursing hospitals. This method was chosen to ensure that participants could provide rich, relevant, and experience-based insights into the phenomenon under study [26].

The number of general nurses was chosen because the senior ward nurses at the institutions mentioned above suggested that nurses with the most experience working with patients with dementia were the most qualified. The interviews were carried out in three cities. All participants were women (100%), with an age range from 29 to 68. The sample sufficiently revealed the topic, and the responses were repeated afterward, achieving data saturation. To determine nurses’ experience in caring for patients with dementia, data saturation was sought. It was decided not to expand the sample further and to limit the number of participants to 9, as the responses of the eighth and ninth participants began to repeat themselves, no new categories or themes emerged, and all the main research questions already had sufficient data. Saturation was assessed not only through the repetition of responses but also at the point at which no new codes, categories, or conceptual insights emerged during iterative analysis. This indicator allowed us to determine whether the sample was sufficient to analyze the phenomenon under study and whether the collected data reflected the diversity and depth of the subjects. The quality and validity of the study depended on data saturation [27].

The selection criteria were nurses working in a supportive treatment and nursing hospital who agreed to participate in the study and were fluent in Lithuanian. Exclusion criteria: nurses without direct dementia-care responsibilities, nurses in exclusively administrative roles, and those on extended leave during the data collection period. These inclusion criteria were essential for analyzing nurses’ experiences of Caring for Patients with Dementia in Supportive Treatment and Nursing Hospitals.

### 2.3. Data Collection

Data were collected through individual semi-structured interviews with participants. To make the study as comprehensive as possible, it was planned to interview nurses from various supportive treatment and nursing hospitals. After obtaining permission from the institutions, senior nurses were contacted, who helped identify nurses working with patients with dementia. After receiving their written consent, a date for the visit to the institution was agreed upon. All nurses were given a patient information form about the study and signed a written consent form to participate. After receiving supervisor AJA’s training on conducting semi-structured interviews, Vilnius University final-year student KA carried out interviews with nurses. The interviews were conducted by KA, who had no prior relationship with the participants. Several strategies were used to minimise potential bias: regular debriefings with AJA, during which preliminary insights and interpretations were discussed; adherence to a prepared interview guide, which ensured consistency and reduced the interviewer’s influence on the direction of the conversation; and maintaining a neutral stance during the interviews, with KA avoiding evaluative comments, non-verbal cues, or questions that could steer participants’ responses. The interviews lasted an average of 35 min. The duration varied because some nurses spoke quickly, while others spoke slowly.

The interview questions were developed based on the study aims and existing literature on dementia care [6,15,16]. It was pilot-tested with five eligible nurses to ensure clarity and relevance, resulting in minor refinements before data collection began.

Before the interview began, participants completed a brief sociodemographic questionnaire including gender, age, education, total years of work experience, and years of experience working with people with dementia. Open-ended questions were divided into three themes: 1. Identifying nursing needs. How do you identify a patient’s needs when they can express them verbally? How do you identify the needs of a patient with dementia when they are unable to express them verbally? 2. Care for people with dementia. Questions focused on nursing actions related to patient safety, the environment, and daily care. What are the main actions you take when caring for patients with dementia? What is your perspective on the need for a safe patient environment, and how should this environment be structured? 3. Patient behavior management and situation management. This group covers questions about managing challenging behavior, experiences, and methods used. Have you encountered situations at work where a patient with dementia became very agitated? What did you do in those situations? Please provide examples. What non-pharmacological methods help to calm the inappropriate behavior of a patient with dementia?

The collected data. The interview recordings were transcribed verbatim into a computer, creating a separate document for each participant for accuracy and order. The respondents’ answers were analyzed, and the most important statements and recurring themes were identified.

Respondents’ answers were recorded using a voice recorder. To protect the confidentiality of the data, the identities of the respondents have been anonymized by assigning them unique codes, and interview quotes that could identify individuals have been removed. The results were used for scientific purposes while protecting the individual’s privacy.

The interviews were transcribed in Lithuanian, and the quotations were translated into English by both members (KA and AJA) for this article. To ensure accuracy, a double-checking procedure rather than literal translation, thereby preserving contextual meaning.

### 2.4. Data Analysis

A qualitative content analysis was undertaken to process the collected data [28]. This method emphasizes the systematic interpretation of meaning, context, and patterns within textual material [29].

The analysis followed the steps of qualitative content analysis described by Graneheim and Lundman [28]. Two researchers independently read all transcripts several times to gain a sense of the whole. They then conducted independent initial coding, identifying meaning units and assigning preliminary codes. After independent coding, the researchers met to compare, discuss, and reconcile differences, reaching consensus on a shared coding scheme. A coding tree was developed iteratively. Codes were grouped into subcategories and categories based on similarities and differences. Qualitative data analysis software was not used. Study participants were assigned identifiers N1–N9.

The collected data was interpreted by identifying similar experiences and opinions, linking different opinions, and tabulating the information. Items relevant to the study were grouped into categories and further subcategorized by assigning statements. The results obtained were interpreted and analyzed as far as possible, including the nurses’ experience working with people with dementia.

#### Trustworthiness

The trustworthiness of the study was ensured using Lincoln and Guba’s criteria [30]. Credibility was supported through prolonged engagement with the data and independent coding by two researchers (AJA and KA). Transferability was enhanced by providing detailed contextual information about the setting, participants, and analytic process. Dependability was ensured by documenting methodological decisions, and confirmability was strengthened by demonstrating that findings were grounded in participants’ accounts rather than researcher bias.

### 2.5. Ethical Consideration

On 17 February 2023, the Ethics Committee of the Department of Nursing, Institute of Health Sciences, Vilnius University granted permission No. KT-40, along with the institutions’ permissions, to conduct the study. The study followed the most important requirements of scientific ethics: respondents provided free, informed consent to participate; confidentiality and anonymity were ensured; detailed information about the study was provided; and the collected data were stored on a USB stick in a safe deposit box.

## 3. Results

The study involved 9 nurses working with dementia patients across 3 hospitals. The interviews were conducted with nine nurses working at three supportive treatment and nursing hospitals across three cities. The respondents’ data were depersonalized by assigning specific codes, and their characteristics are presented in Table 1.

All participants in the study were women. The youngest participant was 29, and the oldest was 68. Most nurses had a postsecondary non-university education (n = 6). The participants’ total years of experience as general nurses ranged from 2 to 49, with 2 to 27 years of experience working specifically with patients with dementia. The nurses’ work shifts varied: five nurses worked the day shift, and four worked various shifts, day and night.

### 3.1. Identifying Nursing Needs

This group included questions about how nurses identify nursing needs, taking into account patients’ communication abilities. Nursing needs of people with dementia when patients were able and unable to express themselves verbally were divided into two categories: physiological needs (subcategory: eating and drinking, personal hygiene, identifying pain) and social needs (subcategory: communication, activities).

Participants described that verbally expressive patients most often articulate basic physiological needs, particularly hunger. Nurses noted that patients frequently request food repeatedly, even after eating, reflecting impaired satiety recognition.

“<…> it is only after they have eaten that they say they want to eat: can I have something to eat, can you give me something to eat?”,

(N8)

“Food is essential to them, you have to make sure they do not eat sweets because they do not realize they have eaten and they want to eat again”.

(N1)

“…tells you when they want to eat, only they eat and forget when they’ve eaten…”.

(N6)

Some participants also mentioned requests related to bathing, toileting, and pain, although pain was often expressed only as a general complaint without clear localization, making assessment challenging.

“<…> tells me that something is wrong, something hurts, I can react faster and act”,

(N2)

“…he says he’s in pain, but he can’t name where it is.”

(N8)

Beyond physiological needs, nurses emphasized the importance of social connection. Patients actively sought communication, emotional closeness, and recognition, often addressing nurses in familial terms. Participants highlighted that understanding a patient’s life history—such as former occupations or hobbies—helped establish rapport and engage them in meaningful activities. For example, former nurses continued to gravitate toward familiar roles and activities within the care environment.

“<…> they remember very well what happened in the past, and you can have a conversation <… > there is a patient, a former nurse, she comes to the door where it says “nurses’ post,” and she wants to help me, I give her a sheet, and she helps me fill it in <…> I introduced her to the student as a former nurse, and she says: “I was not—I am”,

(N1)

People with dementia’s verbal expressions reflected a broader drive to maintain physiological regulation, social identity, and continuity of self, even as cognitive decline progresses.

The most common nursing needs that were not described but are understood or non-verbally expressed by patients with dementia were eating and drinking, and personal hygiene. Nurses identified that when patients wanted to eat, they did not say so; their behavior changes showed this.

“the needs that support life are eating…”

(N2)

“gestures to the glass, looks at it—means wants to drink…”

(N6)

“<…> it is taking it away from someone else, aggression, hitting”

(N9)

Other respondents noted that physiological needs are essential for the patient, while respecting the patient’s past hygiene habits.

“…washing—everything to do with personal hygiene”

(N3)

“…pointing to nappies, means full, wants to change<…> bathing, nail clipping—all that hygiene”

(N6)

When a patient was not able to speak, nurses pointed out that many patients have a heightened need for pain relief, which they are very good at expressing with body language or gestures.

“…often they frown or just scream—I know they are in pain and they need medication.”

(N3)

“…hand points to the throat or head to signify that the patient has a sore throat or headache.”

(N6)

“When it hurts, the facial expressions are like this…”

(N4)

In making these statements during the interviews, the nurses used body language: hand gestures and facial expressions, all of which reinforced their experience in identifying the pain experienced by the patient.

Nurses reported that although patients cannot express their wishes verbally, they still wanted physical contact.

“…they want to communicate—they catch my hand when I do something…”

(N4)

“…wants to make contact, hug—grabs my hand, pulls me close…”

(N9)

Thus, it can be seen that nurses tend to ensure and mostly attend to physiological needs, while not forgetting the importance of social needs, which are essential for people with dementia.

### 3.2. Care for People with Dementia

Questions focused on nursing actions related to patient safety, the environment, and daily care. Participants were asked about the need for a safe environment for the patient and what this environment should look like. All participants in the study identified creating a safe environment as one of the most critical aspects of providing quality services to people with dementia. Factors influencing the creation of a safe environment for patients with dementia were divided into three categories: objects for the prevention of falls (subcategory: good lighting, non-slip surface, safe restraints, suitable footwear), injury prevention objectives (subcategory: no sharp objects, sealed windows), and social safety (subcategory: maintaining patient routines, conscious ward neighbours, and maintaining availability).

During the interviews, nurses identified that a safe environment includes a thoughtful layout of the rooms throughout the hospital and the objects and installations within them—good lighting, non-slip surfaces, and the installation of safe supports.

“<…> a person who is not oriented goes to the toilet, and there they usually slip, fall, or bump themselves. If he gets out of bed and the light comes on from the movement, the risk of falling is already reduced. In the corridors with toilets, I tell them to leave the light on because when someone gets up, they will always go into the light, not the dark”

(N1)

“It’s good to have a support where you can get a grip, but without sharp corners…”

(N8)

“<…> so that there are no chairs or things in the way, so that the floor is not wet, so that they do not slip. They look around, but they cannot see”

(N9)

It was not only the environment itself that was important for a safe environment, but also the patient’s appropriate footwear, which was essential to prevent falls in a patient with already difficult mobility.

“<…> we make sure that the footwear, the tap shoes, are not slippery, and that the patient is protected, otherwise not even the walkers will help”

(N6)

Many nurses stressed that preventing injury or death is crucial in creating a safe environment. The majority of nurses identified the absence of sharp objects as the most important aspect for protecting patients, other patients, staff, and property.

“…because they can cut themselves, and if they get into a fight with another patient, they can use …”

(N2)

“…you need to take away sharp objects, both scissors and knives, because there are suicidal options”

(N3)

“<…> no sharp objects in the ward, because he can pick them up and use them for the wrong purpose <…> so that he is safe, does not injure himself or do something bad to others”

(N8)

“<…> he was here, he started to open the cupboards, he wanted to smash everything, so it is good that he does not have any sharp objects, which would be dangerous for everyone”.

(N6)

Another essential element in ensuring a safe environment was sealed windows. Nurses said that windows should be locked or handleless, and only medical staff should be able to open them if necessary, as there had been cases of patients jumping out of windows.

“Windows need to be watched because demented people can fall out of them.”

(N1)

“windows with locks, because there was a case of someone falling out…”

(N2)

“We do not know what she is thinking; she does not understand why she is here and behaving like this…”

(N4)

Nurses paid attention to the environment and ensured the patient’s care process was as safe as possible. Nurses observed that not only physical things but also social safety were of particular importance.

“If you trace a person’s routine, what they do when they get up to go to the toilet, and you walk them to the toilet, you’ve already solved the problem…”

(N1)

“It is essential to create a daily routine for the patient, not to distract him, because it is already difficult for him to know what is going on, to remember what is going on, to remember what he is supposed to be doing, when to go to bed when to get up when to eat when to wash when to do activities…”

(N8)

Nurses also noted that other patients on the ward could be a safety risk. Thus, dementia patients were better placed in single wards or with other conscious patients to avoid injuries.

“…we try to put them in such a way that there is at least one sensible person on the ward so that they can inform us if the patient is going to do something”

(N6)

“<…> when I am lying with another patient, they put a blanket over her, take care of her, stroke her head. We feared the probe being pulled out, but she takes great care of the ward neighbor”

(N7)

Participants noted that employment provides a safe environment for people because they feel needed, listened to, and safe amongst everyone around them, doing what they did before their illness.

“The environment has an impact, the activities, the music in the afternoon <…> it even seems like some people cannot move around like that, but they do move and dance, and it provides a safe environment”

(N1)

It can be assumed that nurses identify a safe environment for dementia patients based on their own experience, emphasizing both the physical factors of the environment and the preservation of patients’ positive emotions.

Participants were asked to list their most common actions when caring for people with dementia. The participants’ actions were divided into two categories: physiological (subcategories: personal hygiene, eating and drinking, mobility, administration of medicines, pressure sore care, risk assessment for falls, patient restraint) and social (subcategories: communication).

All nurses emphasized that the most frequent actions they perform were those that meet the patient’s physiological needs, particularly personal hygiene. During the interviews, the nurses’ responses were repeated, stating that the patient is washed and bathed, that diapers are changed, that the urinary catheter is cared for, and that nails are clipped.

“…changing diapers, bathing—everything to do with hygiene.”

(N2)

“…time to change diapers—we changed them. Then it’s time for bathing—we took a bath, cut the nails, the whole routine of physiology.”

(N5)

“<…> personal hygiene—bathing, changing nappies, and also taking care of the urinary catheters” (N4), “<…> personal hygiene, we do contribute a lot to that: washing, bathing, changing nappies, meeting his/her physiological needs”

(N6)

Another action identified by the study participants was ensuring that the patient was fed and had drinks. This action was significant as dementia patients were prone to choking. The nurse’s vigilance was reflected in the interviews.

“<…> dementia patients can choke <…> we all try to eat at the same table, and the ones who cannot walk go round in a wheelchair, we are just like one big family”

(N1)

Several nurses emphasized the importance of feeding and monitoring diet, as people with dementia not only forget that they have eaten but also often move around a lot.

“<…> there is a loss of weight because those who walk a lot need to be monitored, and we introduce a diet of protein to keep the balance”

(N1)

“<…> you have to watch the diet…”

(N9)

Participants also identified medication administration as an integral part of dementia care.

“<…> medication in the morning, at lunch, in the evening…”

(N2)

“…monitoring of the medication to see if they take it.”

(N6)

“…they take their medication, I rub it, because if it’s a bigger tablet, they won’t drink it, they don’t understand what’s there”

(N7)

Two participants in the study identified the importance of movement for patients in the more severe stage of the disease. Although physiotherapists focused on patient movement and exercise, nurses noted that they must also encourage patient movement.

“…it doesn’t matter that it’s a lying person, but with us all the patients are lifted <…> health and circulation is needed.”

(N1)

“<…> We give patients walkers and encourage them to move, so we always make sure they do not fall, but they are often happy because they see something new, they just change the ward environment”.

(N6)

Ensuring movement was also essential to prevent pressure sores, and if the patient’s lying position remained unchanged, nurses had the additional task of providing pressure sore care. The nurse noted that the patient’s low mobility could lead to pressure sores. Also, due to the imbalance, assessing the risk of falls was necessary. Several nurses noted that one of the nursing actions was the fixation of the dementia patient to the bed due to the inability to communicate and aggressive behavior.

“<…> it happens that we tie their hands to prevent them from pulling away <…>, of course, there are all the consents, we let go every once in a while <…> it is scary to tie someone up, but there is no option”

(N4)

“…we fix the leg for the night, because if you don’t fix it, you put it down and it gets up, it goes away.”

(N7)

Fewer nurses also reported carrying out activities to meet social needs. Participants found time to interact with the patient.

“<…> People are often weighed and measured everywhere, but there is no emotional side, so I try to welcome them, reassure them, and get to know them, because it is a new place for them and can be stressful. I ask about the past, what he liked, how many children he has…”

(N1)

“They feel the other person’s emotions when they are angry with him. That is why it is essential to communicate nicely with them, not to raise your voice, to try to gain their trust”

(N8)

All the nurses emphasized that the majority of their work involved attending to physiological needs, particularly personal hygiene. The specific tasks most frequently mentioned during the interviews were: washing and bathing patients, changing diapers, caring for urinary catheters, and trimming nails. A much smaller proportion of participants reported systematically engaging in activities to meet social needs. The nurses stated that, when possible, they set aside time to interact with the patient.

### 3.3. Patient Behavior Management and Situation Management

The follow-up interviews aimed to determine whether there were any situations in which the dementia patient became very nervous and what the nurses did in response. All nurses reported encountering situations in which the patient became nervous or aggressive. Nurses identified the actions they take to calm down an aggressive patient, both on their own and with the team’s help. Nurses’ actions in situations of inadequate patient behaviour were divided into two categories: in person (subcategories: quiet conversation, diverting attention, patient isolation) and in the team (subcategories: medication administration, patient restraint).

When a patient becomes aggressive, nurses try to talk to the patient calmly. Most nurses tried to calm the agitated patient with words, to stay calm, and to speak.

“<…> first of all, to ask nicely to hand over the object, not to grab the hand and say “give it back”

(N1)

“<…> first of all, to communicate calmly, because if you raise the tone or give orders, instructions, it can affect them badly, but if you are calm, nice, it calms them down”

(N2)

“…of course we are trying to talk, to calm…”

(N4)

“to speak gently and nicely, and then no anger <…> in a language they understand.”

(N7)

Another way to calm an aggressive patient was to redirect attention. Nurses said distraction was an effective option because dementia patients often forget. However, the distraction should be in a direction the patient likes to achieve the maximum calming effect.

“…says he needs to go to the sauna, he needs to go to X street, so we say you can go, and then we take him to watch TV, he calms down…”

(N4)

“It is essential to know what the person likes, to offer them other activities <…> to divert the attention and say let us go and look for the way home and take them to the table, and maybe the person likes drawing or doing a jigsaw puzzle and offers them to do that <…> they forget what they were doing just now very quickly”

(N8)

Nurses also tried to isolate the patient. The participants in the study said they try to leave the patient alone when conditions are right, which helps calm the patient and protects all staff and other patients from the dementia patient’s inadequate, unpredictable, and potentially dangerous actions.

“…but we provide a safe environment for the other patients as well as for ourselves by leaving him alone…”

(N5)

“<…> we leave the patient alone in the ward, he is screaming there, but we provide a safe environment for the patients and ourselves by leaving the patient alone <…> if it is a safe environment, it is to let the patient calm down, and we need to have no more patients on the ward”

(N8)

Nurses identified that after an unsuccessful interview, they took medication in collaboration with doctors.

“<…> I take appointments, and together with the psychiatrist, with his permission, we increase the dosage of certain medications”

(N2)

“Most of the time, we call the on-call doctor, the psychologist, and if necessary, the psychiatrist. They prescribe some medications”

(N6)

“If she becomes aggressive, starts hitting others, we look for a sedative prescribed by a doctor.”

(N9)

Several nurses said that in the case of a very aggressive patient, it was necessary to take quick action and fix the patient for the safety of all.

“It is good to be able to strap the patient across the abdomen with these straps, because when it is just the arms he can get away, and here it is more difficult when it is across the abdomen”

(N4)

“The psychiatrist gives permission to fix the patient, then for the safety of the patient, so that he does not hurt himself, does not break anything, does not injure someone else…”

(N6)

The interviews revealed that all nurses encounter episodes of anxiety and aggression among patients with dementia. The interventions they use were divided into individual (calm conversation, distraction, patient isolation) and team-based (medication administration, physical restraint of the patient).

Calm communication was usually the first approach used to reduce the patient’s tension. If this was ineffective, nurses redirect the patient’s attention to activities they enjoy or, if necessary, temporarily isolate the patient in a safe environment. If the situation could not be managed with non-pharmacological measures, medication was adjusted in consultation with physicians. In high-risk cases, patient restraint was used with the physician’s permission to ensure everyone’s safety.

Nurses were asked to list the non-medication methods that help to calm the inappropriate behavior of a dementia patient. Nurses’ actions to reduce aggressiveness without medication were divided into six categories: redirecting attention, physical contact with the patient, conversation, patient isolation, involving relatives, and walking.

The participants tried to distract the dementia patient to calm him down. Nurses diverted the patient’s thoughts with things usually in the hospital—TV, plants, clothes, compliments, and other surroundings.

“You can distract him somewhere by knowing what he likes and does not like <…> invite him to look at some flowers, praise him for dressing so nicely, how nice he looks”

(N2)

“We turn on the radio, the TV, bring make-up, powder <…> because they stay with us for a long time, we can see what they like.”

(N5)

“There is that they like to paint, so we bring paint”

(N6)

“<…> sit him in front of the TV, give him a grandmother’s old man to push around with a wheelchair, who drives it, to make him calmer…”

(N7)

“<…> diverting thoughts to other activities—dancing, food—we offer to drink tea and immediately calm down”

(N9)

Several nurses say that they tried to talk to the patient.

“…first you try to calm the person down, to take them to another environment and talk to them there.”

(N1)

“Talking, singing together…”

(N3)

“It depends on the patient, but I think in any case you have to talk to the patient, or you do not talk to the patient, you try to talk to the patient, and maybe they will tell you why they are reacting the way they are reacting”

(N4)

During the interview, nurses identified gentle touches to try to reassure the patient.

“…you can hug that person or touch their arm, no grabbing, no pulling…”

(N8)

“<…> we hug them, we hold their hand and pat them, it is like a non-verbal way of saying that we wish them well…”

(N9)

One nurse stated that allowing the patient to be in a quiet area was an effective way to quell inappropriate behavior.

“You leave them alone in that room <…> of course, after a while, in the observation position, you come in, and usually after fifteen minutes the person is calm…”

(N1)

Several participants in the study stressed that relatives also needed to take the initiative to calm the patient, as nurses did not have much time to attend to everyone.

“<…> they would talk so that they would feel the attention not only from us but also from the household.”

(N6)

“<…> if they do not calm down, I say I will call the relatives…”

(N9)

One nurse thought that walking was also a non-medicated way to calm down because it changes the patient’s environment.

“<…> a walk in the fresh air brightens up the day, allows patients who are in the same ward every day to relax from the stress”

(N9)

Nurses identified several non-pharmacological measures that helped manage inappropriate behavior in patients with dementia. The most commonly used methods include: redirecting attention, calm conversation, gentle physical contact, temporary isolation, involving relatives, and taking the patient for a walk. These measures often help reduce the patient’s anxiety, but they are not always sufficiently effective.

## 4. Discussion

The survey participants’ experiences were compared with those of other authors’ studies to reveal the unique experiences of nurses in a global context. Each experience is unique, shaped by the nurse’s approach and the relationship they build with the dementia patient. Person-centered care is a holistic, integrated approach that aims to ensure the well-being and quality of life of people with dementia by involving the individual, their caregivers, and their family in their care [31]. Fazio et al. (2018) described person-centered care as a philosophy of care based on the individual’s needs and on how well one knows that person, with a strong interpersonal relationship established [32].

Ensuring the care needs of people with dementia are met is a priority. As dementia is a progressive disease, the care needs of the person with dementia change with each stage of the disease. Nurses in the study identified that regardless of the stage of the patient’s illness, it is essential to know the patient’s life before the disease, as most of the patient’s needs are related to their past. Insight into the patient’s past life is also highlighted as necessary for providing quality care in other studies [13,14,33]. Besides, Du Toit, Shen, and McGrath (2019) found that occupational deprivation persists and person-centered care is not fully achieved when opportunities for growth and engagement for residents with moderate to advanced dementia do not extend beyond simply acknowledging their life history [34].

The primary care needs of people with dementia identified by the participants were eating and drinking, personal hygiene, pain management, communication or physical contact, and activities. Other studies also reflected these needs, but with the addition of spiritual needs, the ability to express faith and prayers [8,35]. Schmidt et al. (2018) found that people typically express their religiosity either individually or within a community through religious exclamations, songs, or prayers before meals [8]. Some residents kept religious objects, and the interior of their personal rooms reflected religious themes [8].

A safe environment is ensured by providing certain conditions, such as safe rooms, removal of sharp objects, tight window locks, maintenance of the patient’s routine, and conscious ward neighbors. Nilsson et al. (2019) emphasized that safety is particularly crucial for care recipients with cognitive impairment, who face a higher risk of falling or wandering because they struggle to navigate unfamiliar environments [36]. Another study showed that nurses struggled with the ward’s physical design and layout when trying to create a more familiar environment to reduce responsive behaviours. This challenge was particularly evident in wards where people with dementia shared a room with other patients [7].

The analysis of nurses’ actions when caring for people with dementia highlighted actions that meet physiological and social needs as part of nurses’ daily routines. Our nurses’ emphasis on personal hygiene, eating and drinking, and administering medications to patients with dementia is reflected in other authors’ studies of the advanced stage of dementia [8,37].

The experience of the nurses we interviewed differs from others in how they meet the patient’s social needs, with less time spent on socializing and activities. However, nurses identify this as essential and understand it [33]. In the broader practice of patient-centered nursing by foreign nurses, the emphasis on connection with the patient shapes the distribution of nurses’ actions, with a focus on improving the patient’s emotional state [38]. Much attention has been paid to analyzing nurses’ responses to inappropriate patient behavior. Without a precise algorithm, nurses rely on their own experience: removing environmental stimuli, talking calmly to the patient and redirecting their attention, administering medications according to the physician’s recommendations, and, if necessary, fixing the patient. Foreign authors have noted the same actions, but fixation of the patient is usually avoided by finding the reasons that may have triggered the behavior [16,39].

When caring for people with dementia, physiological needs are often prioritised over social needs because patients with dementia frequently require continuous assistance with eating, drinking, hygiene, mobility, and safety. Such care is highly time-consuming, and meeting physiological needs is essential for maintaining basic functioning, which leaves limited opportunities for social engagement. As cognitive decline progresses, patients’ ability to participate in social activities also diminishes, further shifting the focus of care toward meeting immediate physiological needs. Besides, participants describe the use of restraint or isolation as a measure applied only as a last resort, when all other de-escalation strategies have been exhausted, and the patient poses an immediate risk to themselves or others. Nurses emphasised that such actions require clear criteria, physician authorization, and continuous observation to ensure patient safety. They also noted the importance of documenting the circumstances, duration, and rationale for restraint, followed by a review of the incident.

### Study Limitations

The study has certain limitations. First, the sample size was small, suggesting the data may not reflect the experiences of the broader nursing population. In addition, only women participated in the study, limiting the ability to examine gender differences in nursing experiences. Participants were identified by senior ward nurses, who selected those they regarded as the most experienced. This may have led to a more homogeneous perspective, dominated by highly experienced staff. Future studies should also include less experienced nurses to capture a broader range of perspectives. There is also a risk of social desirability bias—participants may have provided responses that were more socially acceptable rather than fully reflecting their actual experiences. Finally, the interviews were conducted by a student researcher, which may have influenced participants’ openness or the depth of their responses, particularly if she was perceived as less experienced. Besides, this study also has limitations related to the use of a semi-structured interview guide. Because the questions were designed to elicit descriptions of nurses’ experiences, the guide may have shaped the predominantly descriptive nature of the findings. The structure of the interview prompts could have directed participants toward certain aspects of care, potentially limiting the emergence of more interpretive or unexpected themes.

## 5. Conclusions

The study’s findings highlight the diverse and evolving care needs of people living with dementia and the central role nurses play in meeting these needs. The needs of dementia patients highlighted by the nurses in the study varied according to each patient’s condition. These needs included adequate food and fluid intake, personal hygiene, freedom from pain, communication with nurses and other patients, and meaningful activities. For patients unable to communicate verbally, essential needs were eating and drinking, personal hygiene, pain control, and physical contact with nurses.

The most common activities nurses performed with dementia patients were bathing, changing nappies, clipping nails, ensuring adequate nutrition, and preventing choking. Nurses also paid close attention to medication administration, including applying topical treatments and ensuring that medicines were taken. Nursing staff encouraged mobility as much as possible to prevent pressure sores and used bedside restraints when necessary. Restraints were applied, and the decision-making process and institutional protocols were intended to ensure patient safety and uphold ethical standards. Communication and organizing meaningful activities were also part of nursing care, but these were not always achieved due to limited time.

The challenges nurses face, such as environmental constraints, limited opportunities for activity, and the need to manage responsive behaviours, indicate a need for organizational policies that prioritize dementia friendly environments, interdisciplinary collaboration, and non-pharmacological approaches to behavioural symptoms. Strengthening these areas in practice and policy would support more holistic, person-centered care and improve the overall quality of life for individuals with dementia.

## Figures and Tables

**Table 1:** Participants were distributed by gender, age, education, total years of work experience, and years of experience working with people with dementia.

Respondent (Code)	Gender	Age (in Years)	Education	Total Years of Work Experience	Years of Experience Working with People with Dementia
N1	female	43	Higher non-university education	12	12
N2	female	68	Higher non-university education	49	28
N3	female	32	Higher non-university education	9	9
N4	female	62	Higher non-university education	40	20
N5	female	49	Higher education	27	27
N6	female	31	Higher non-university education	7	7
N7	female	64	Higher education	41	20
N8	female	52	Higher university education	28	2
N9	female	29	Higher non-university education	2	2

## Data Availability

The data presented in this study are available on request from the corresponding author due to ethical restrictions.

## Public Involvement Statement

No public involvement in any aspect of this research.

## Guidelines and Standards Statement

This manuscript was drafted against the COREQ (Consolidated criteria for reporting qualitative research) for qualitative research.
